# Study on Creep Damage of Ni-Based Superalloy Caused by Variable Load Conditions at Elevated Temperatures

**DOI:** 10.3390/ma14226971

**Published:** 2021-11-18

**Authors:** Sunguk Wee, Keekeun Kim, Kibum Park, Changsung Seok

**Affiliations:** Department of Mechanical Engineering, Sungkyunkwan University, Suwon 16419, Korea; sinkill2@naver.com (S.W.); rlrms1204@naver.com (K.K.); rlqja0711@naver.com (K.P.)

**Keywords:** low-cycle fatigue, creep, CMSX-4, Ni-based superalloy

## Abstract

Higher fatigue and creep resistance at high temperatures are the essential properties for materials such as those used in gas turbines for power generation and aircraft turbines. Therefore, the nickel-based superalloy CMSX-4 was developed through single-crystal casting to satisfy these requirements. In this study, the CMSX-4 creep test results reported by previous researchers were used to mathematically derive an equation to estimate the amount of creep damage occurring under variable load conditions. In addition, low-cycle fatigue tests were performed, and the effect of creep damage occurring during fatigue on material failure was described.

## 1. Introduction

**Figure 1:**
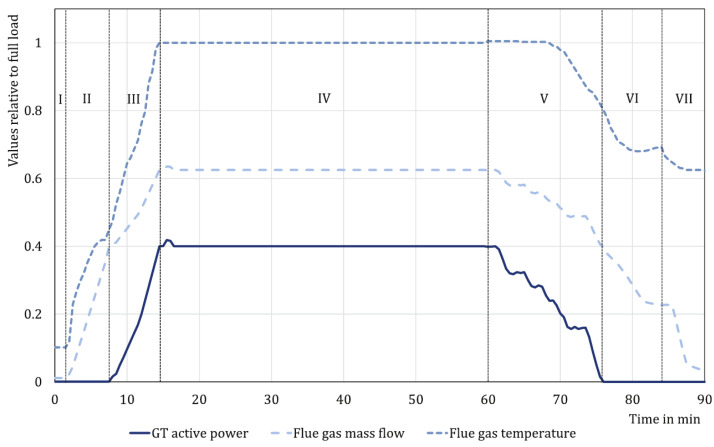
Applied load with respect to the operating time graph (I–III Start up; IV steady state; V–VII shut down) [11].

Ni-based superalloys have superior properties, such as high creep resistance at high temperatures, compared to other metals, including steel, aluminum, and titanium. Therefore, they are used primarily as materials for parts used at high temperatures, such as aircraft engines and gas turbines for power generation [1,2,3,4]. In general, a turbine is operated at a high temperature, and a load is added through centrifugal force during the operation [5]. Therefore, the material used for the turbine is exposed to creep and fatigue [6,7,8,9,10]. For the analysis of a load applied to a turbine, the load of the turbine with respect to the time occurring in one cycle is shown in Figure 1 [11]. In Sections 1–3, the mechanical load was applied as the temperature increased, and the speed of the turbine increased. In Section 4, the rotational speed of the turbine reached its maximum value, after which it maintained a steady state. Finally, in Sections 5–7, the operation was stopped, the temperature was decreased, and the mechanical load was removed simultaneously. The materials composing the turbine were damaged by fatigue due to the increase and decrease in the mechanical load in Sections 1–3 and 5–7. In Section 4, they were damaged by creep due to the high temperature with a constant load. Therefore, many studies have been conducted on fatigue and creep to predict the life of turbine blades. Biao Ding et al. conducted a low-cycle fatigue test of the Ni-based superalloy DZ445, predicted energy-based fatigue life, and analyzed the microstructure difference when tensile or compressive stress was dominant [12]. D. Holländer et al. observed the difference in the residual fatigue life by performing fatigue life evaluation for the root and airfoil parts of a service-exposed gas turbine blade [13]. G. Marahleh et al. evaluated the creep life of gas turbine blades using LMP and observed the microstructure change [14]. Shuning Gu et al. and Chengjiang Zhang et al. conducted a study on the difference in creep characteristics caused by the difference in the casting process [15,16]. Jinbin Chen et al. analyzed the difference in the creep property of a nickel-based superalloy according to the chemical composition [17]. Although creep and fatigue occur simultaneously during turbine operation, previous studies have assessed creep and fatigue independently. Therefore, some researchers have conducted research by applying dwell time to consider creep and fatigue at the same time. Siyuan CHEN et al. and Biao Ding et al. studied the fatigue life according to the change in dwell time and found that the effect of compression dwell was greater compared to tensile dwell and compression dwell [18,19]. R.J. Kashinga et al. analyzed the difference in fatigue life according to the direction of solidification and the direction of load through a fatigue test with dwell time applied to the CM247DS material cast by unidirectional solidification [20]. However, fatigue studies applying dwell time have limitations because they focus only on the effect of creep occurring in the steady state (IV). Jeong-Min Lee et al. performed a low-cycle fatigue test with different test speeds under the same temperature and strain conditions. As a result of the fatigue test, the fatigue life was measured to be shorter under the condition of a slow test speed than under the condition of a fast test speed. The reason is that slower testing increases creep damage due to longer exposure to high temperatures [21]. Therefore, in Sections I–III and V–VII, damage due to low-cycle fatigue mainly occurs, but creep damage also occurs due to the high temperature and load conditions, meaning both creep and fatigue effects must be considered. For accurate turbine life prediction, it is important to quantify the damage caused by creep during low-cycle fatigue. Therefore, in this study, a low-cycle fatigue test was performed to derive the quantification of creep damage occurring under variable load conditions, and the stress variation over time was derived. In addition, an equation that can quantitatively derive the amount of creep damage from the creep and low-cycle fatigue test results was proposed. By applying the proposed equation to the low-cycle fatigue test results, the amount of creep damage that occurred per cycle was derived, and the effect on the low-cycle fatigue life was analyzed.

## 2. Low-Cycle Fatigue Test

**Figure 2:**
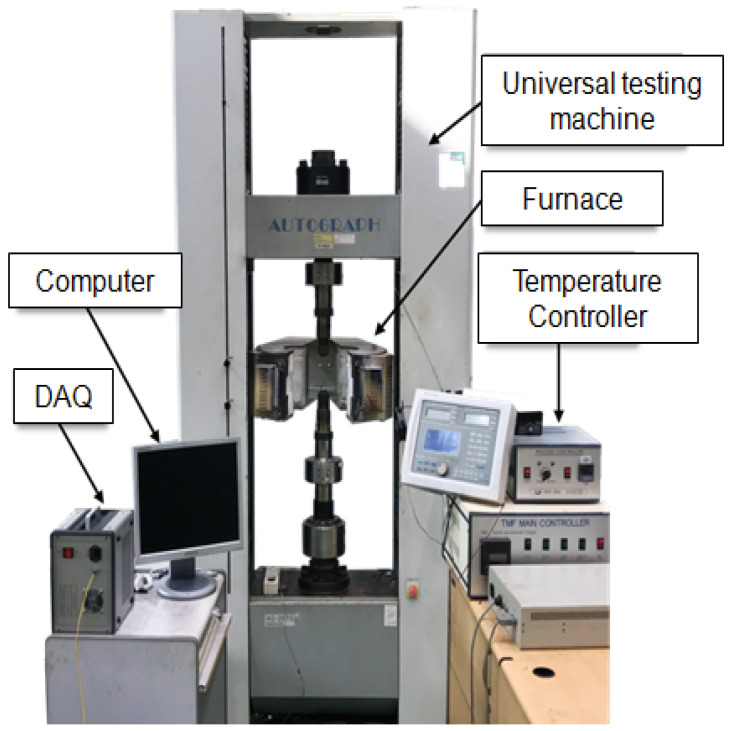
Equipment for the low-cycle fatigue test.

To confirm the effect of creep under variable load conditions, a low-cycle fatigue test, in which tension and compression were repeated at a constant temperature, was performed. For the low-cycle fatigue test, Ni-based single-crystal superalloy CMSX-4 with excellent creep resistance at high temperatures was used. The crystal growth direction of CMSX-4 used in this test was [0, 0, 1], and its chemical composition is shown in Table 1. The length of the parallel portion, the gage length, and the diameter of the specimen were 22 mm, 12.5 mm, and 8 mm. To evaluate the low-cycle fatigue life of CMSX-4 at high temperatures, tests were performed at 800 °C and 900 °C under three strain conditions, as shown in Table 2. Before performing the low-cycle fatigue test, the temperature was maintained for 3 h after the specimen reached the target temperature so that the specimen could sufficiently reach thermal equilibrium. For temperature measurement, the temperature of the parallel part of the specimen was measured using a K-type thermocouple. A 25-ton capacity electric motor-type tester and furnace (1300 °C) were used as test equipment, as shown in Figure 2. The load and strain with respect to the change in time of the low-cycle fatigue test were measured, and the fatigue life was based on the failure of the specimen.

**Figure 3:**
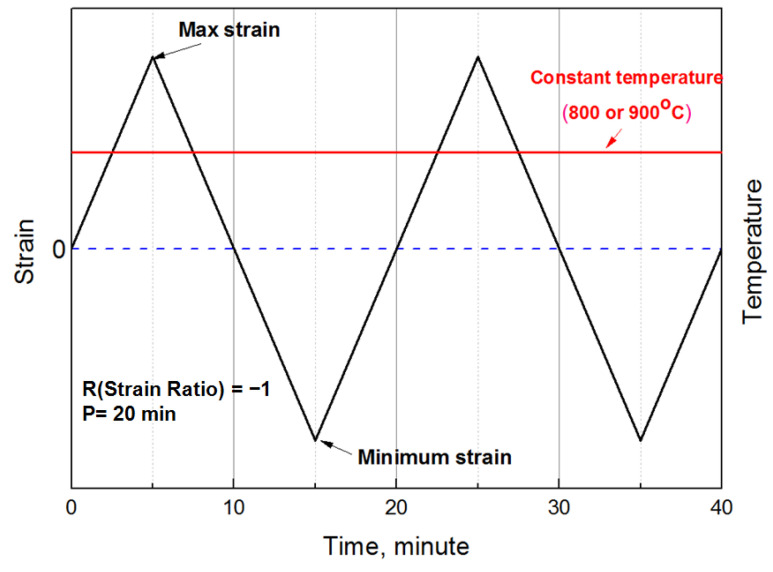
Strain test condition during the low cycle.

**Figure 4:**
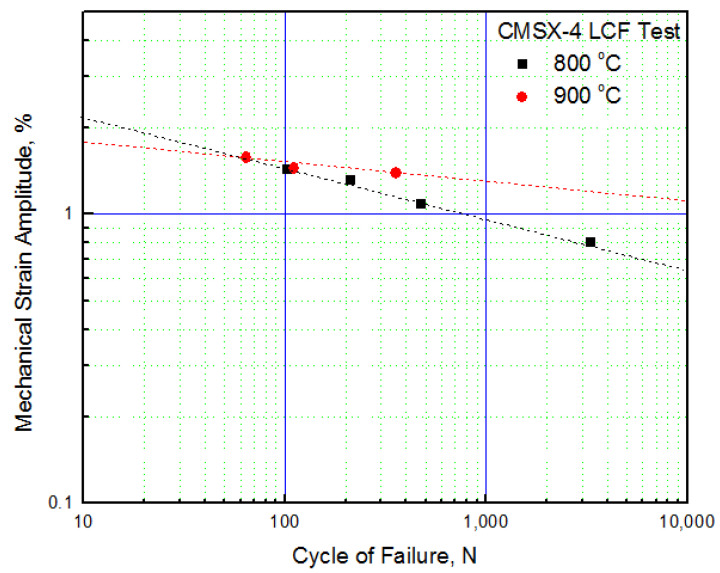
Strain–failure cycle curve (CMSX-4).

Figure 3 demonstrates the test method used to minimize the effect of creep in the steady state. A strain ratio of R = −1 was applied without any dwell time. In addition, a test was performed where one cycle was 20 min, considering the operating environment of the turbine. From the low-cycle fatigue test results, the strain–fatigue curve diagram was derived, as shown in Figure 4. Because the low-cycle fatigue test performed in this study was conducted at a high temperature rather than at room temperature, creep occurred in the section where the tensile load was applied, in addition to pure mechanical fatigue. Therefore, we intended to derive the amount of creep damage that occurred during fatigue using the low-cycle fatigue test results.

## 3. Derivation of Creep Damage Caused by Variable Load

**Figure 5:**
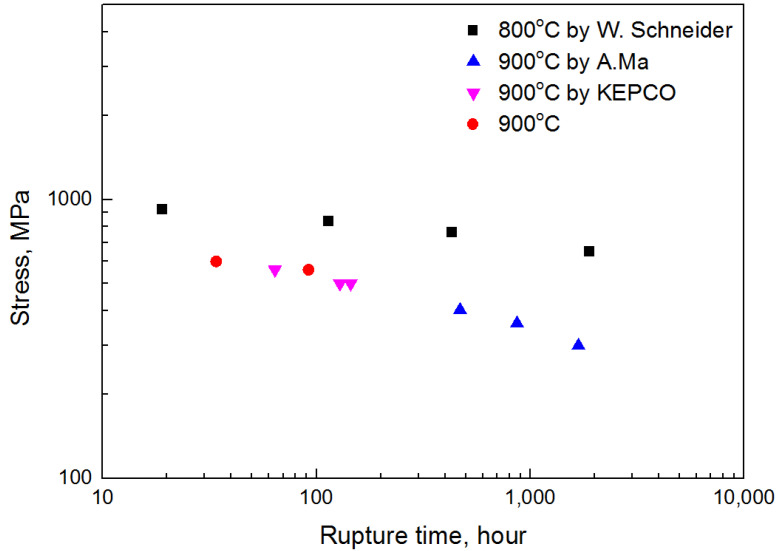
Stress–rupture time graph at 800 °C and 900 °C [25,26,27].

In general, when creep proceeds, the rupture time exhibits a nonlinear relationship with stress. Norton proposed the relationship between rupture time and stress in the form of an exponential function of stress as follows [22,23,24]:

t_r_ = Aσ^n^ (1)

where t_r_ is the rupture time, and A and n are the experimental constants determined experimentally. To evaluate the creep characteristics with respect to temperature, data at various temperatures and stress test data are required. In this study, rupture times of 92 h and 34 h were obtained by performing creep tests at 900 °C–560 MPa and 900 °C–600 MPa, respectively. However, since it was insufficient to show the relationship between creep rupture life and stress, more creep test results were needed. Therefore, we referred to the CMSX-4 creep test results obtained in another study at 900 °C [25,26]. Similarly, to derive the amount of creep damage at 800 °C that occurred during fatigue, W. Schneider’s creep test results at 800 °C were referred to [27]. All creep test results are shown in the stress–rupture time graph in Figure 5. To derive the constants A and n from Figure 5, the relationship between stress and rupture time was fitted using Equation (1) and the least squares method. The values of A and n at each temperature derived from the fitting results are shown in Table 3.

**Figure 6:**
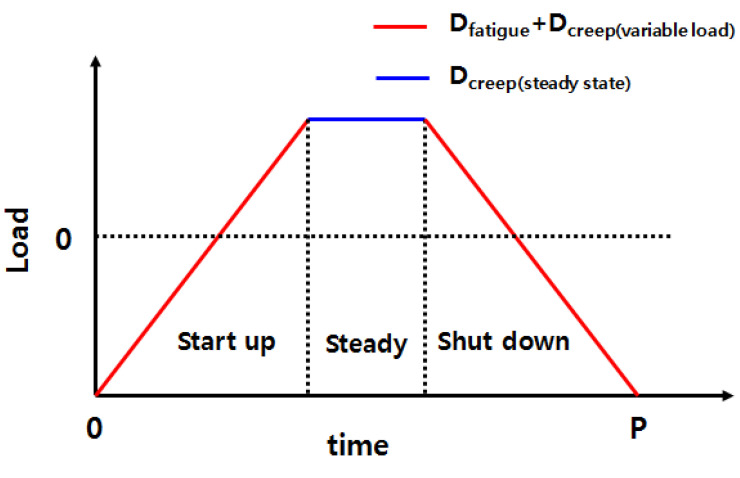
Types of damage with respect to the operating conditions.

In this study, the amount of damage owing to creep occurring during fatigue was derived by applying the cumulative damage law from the relationship between the creep rupture life and stress and temperature derived earlier. Equation (2) represents the linear cumulative damage law, which is used when considering fatigue and creep simultaneously [28,29,30]:

(2) $$\sum D_{\mathrm{fatigue}}+\sum D_{\mathrm{creep}}=1$$

where D_fatigue_ represents the fatigue damage per cycle, D_creep_ represents the creep damage per cycle, and failure occurs when the sum of the two damage amounts is 1. To analyze the damage occurring during the turbine operation, the types and sections of damage under the operating conditions are shown in Figure 6. Here, D_creep_ can be expressed as the sum of D_creep_ (variable load) and D_creep_ (steady state). However, as this study only considered the amount of creep damage that occurred under variable load conditions, it was assumed that D_creep_ (steady state) = 0.

Considering that t_r_ is the creep rupture time at a specific temperature and stress, 1/t_r_, which is the reciprocal of the rupture time, becomes the creep damage received per unit time. Therefore, if the creep damage received per unit time is integrated with time in the cycle range (P) corresponding to one cycle in the fatigue test, the amount of creep damage occurring in the fatigue test per cycle can be expressed as Equation (3). By substituting Equation (1), which is the relational expression between the fracture time and stress, it can be rearranged to obtain Equation (4) as follows:

(3) $$D_{\mathrm{creep}}=\int_{0}^{P}\frac{1}{t_{r}}\mathrm{dt}$$

(4) $$D_{\mathrm{creep}}=\int_{0}^{P}\frac{1}{{A\sigma }^{n}}\mathrm{dt}$$

**Figure 7:**
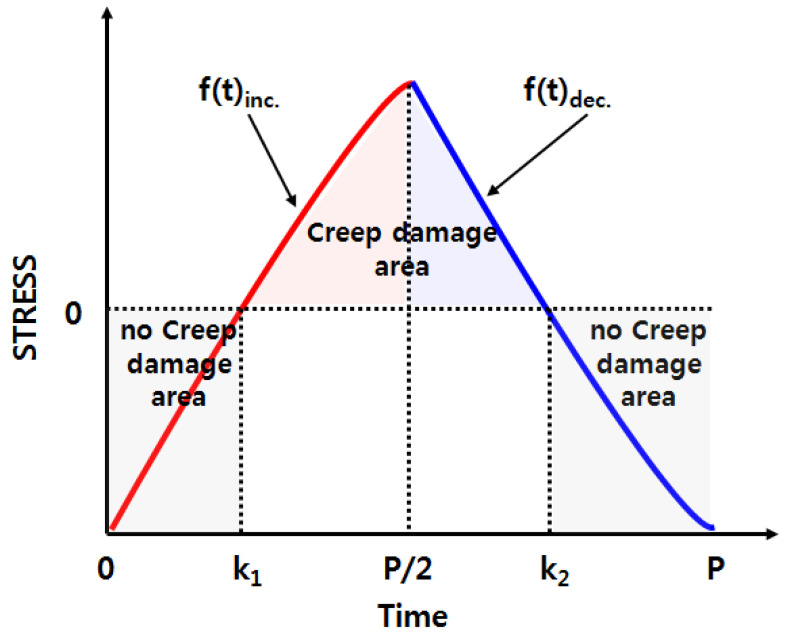
Creep damage area with respect to load conditions.

One cycle of low-cycle fatigue can be divided into tension and compression, as shown in Figure 7. The tensile load was applied up to P/2 (1/6 h), which is half of the entire cycle, and the compressive load was applied up to P (1/3 h). Therefore, to represent stress as a function of time accurately, it was divided into sections receiving tensile and compressive loads based on the P/2 point. The change in stress in the sections where the tensile and compressive loads were applied is defined as a function of time, f(t)_inc._ and f(t)_dec._, respectively. Therefore, Equation (4) can be expressed as Equation (5) based on the integral section, which indicates the amount of creep damage occurring in low-cycle fatigue per cycle.

(5) $$D_{\mathrm{creep}}=\int_{0}^{\frac{P}{2}}\frac{1}{A{\left(f{\left(t\right)}_{\mathrm{inc}.}\right)}^{n}}\mathrm{dt}+\int_{\frac{P}{2}}^{P}\frac{1}{A{\left(f{\left(t\right)}_{\mathrm{dec}.}\right)}^{n}}\mathrm{dt}$$

Creep is caused by the temperature and load; however, as the R (strain ratio) of the low-cycle fatigue test in this study was −1, there was a section where the stress became negative. Therefore, the integral section, shown in Equation (5), and the section where the actual applied creep occurred exhibit a difference. To solve this problem, the points at which the stress became zero (zero point) in the tensile and compression sections were defined as k_1_ and k_2_, respectively. Therefore, in actual low-cycle fatigue, the sections where creep occurs are k_1_-P/2 and P/2-k_2_, and the amount of damage caused by the creep that occurs per cycle can be expressed as

(6) $$D_{\mathrm{creep}}=\int_{k_{1}}^{\frac{P}{2}}\frac{1}{A{\left(f{\left(t\right)}_{\mathrm{inc}.}\right)}^{n}}\mathrm{dt}+\int_{\frac{P}{2}}^{k_{2}}\frac{1}{A{\left(f{\left(t\right)}_{\mathrm{dec}.}\right)}^{n}}\mathrm{dt}$$

## 4. Derivation of Creep Damage That Occurs during Low-Cycle Fatigue

**Figure 8:**
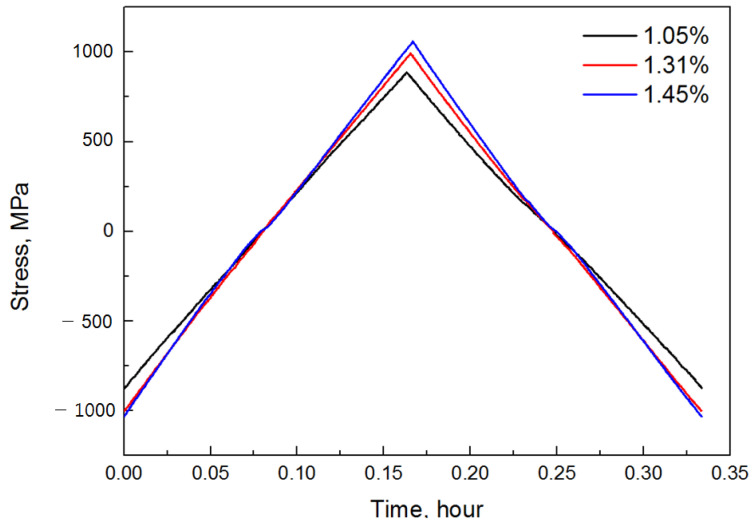
Variation in stress with time at 800 °C.

**Figure 9:**
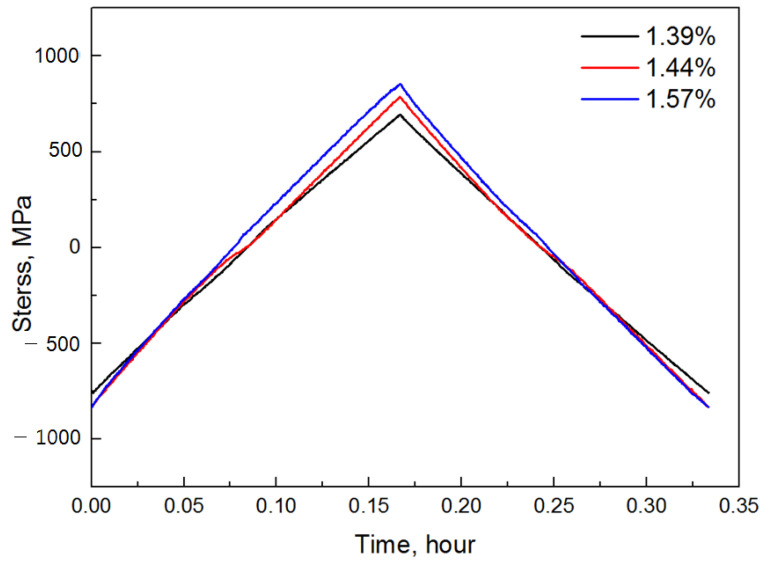
Variation in stress with time at 900 °C.

The low-cycle fatigue test performed in this study took 20 min per cycle; thus, P = 1/3 h. The material constants A and n, which change with temperature, had constant values because the test was performed at constant temperatures (800 °C, 900 °C). However, in the case of stress, the value changes with time; therefore, to integrate Equation (6), stress must be expressed as a function of time. Because the initial tensile section of the low-cycle fatigue test is an elastic section, time and stress are directly proportional to Hooke’s law. However, in the elastoplastic region, time and stress are not proportional. Therefore, the stress–time hysteresis curve obtained from the results of the low-cycle fatigue test described in Chapter 2 (performed at 800 °C and 900 °C) was required to represent the relationship between time and stress accurately. To derive the amount of damage caused by creep, a hysteresis curve at the time of stabilization corresponding to half the fracture life at each strain was analyzed, as shown in Figure 8 and Figure 9.

In general, metals show nonlinear behavior from the plastic region following the increase in stress with time in the elastic region. Therefore, the first and second polynomials and exponential formulas were judged to be capable of simulating the corresponding behavior to each stress–time diagram. Accordingly, Table 4 lists each function’s average values of the coefficients of determination. After a comparison of the coefficient of determination, its average value was found to be high in the order of the first equation, second expression, and exponential function. Therefore, it is most suitable to express the function of stress over time as an exponential function. However, it is not necessary to always use the exponential function because the fatigue hysteresis curve may appear differently based on the properties of the material to be analyzed or the test conditions. For example, in the case of a high strain, a nonlinear section may appear, largely owing to an increase in the plastic region; in the case of a low strain within an elastic section, a simple linear relationship may appear. Therefore, any function expression can be used if it can accurately represent the stress over time.

The stress relational expressions f(t)_inc._ and f(t)_dec._, with respect to time in the tensile and compression sections, were derived using the exponential function. After substituting them in Equation (6), the amount of creep damage D_creep_ occurring per cycle was obtained, as shown in Table 5. As a result of analyzing the amount of creep damage according to the strain, the amount of creep damage occurring per cycle increased as the strain and temperature increased. This is consistent with the general creep phenomenon, in which the fracture life becomes shorter with an increase in stress and temperature [31,32].

## 5. Discussion

**Figure 10:**
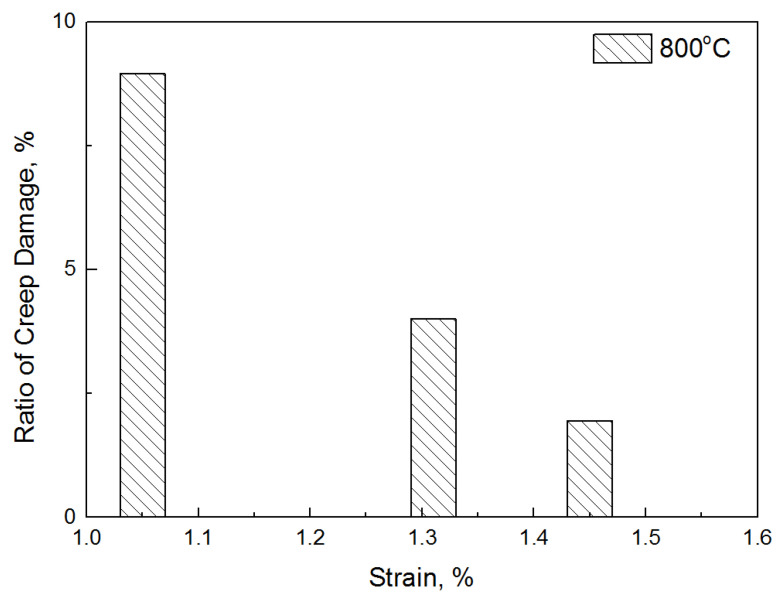
Ratio of damage caused by creep (ΣD_creep_) at 800°C.

**Figure 11:**
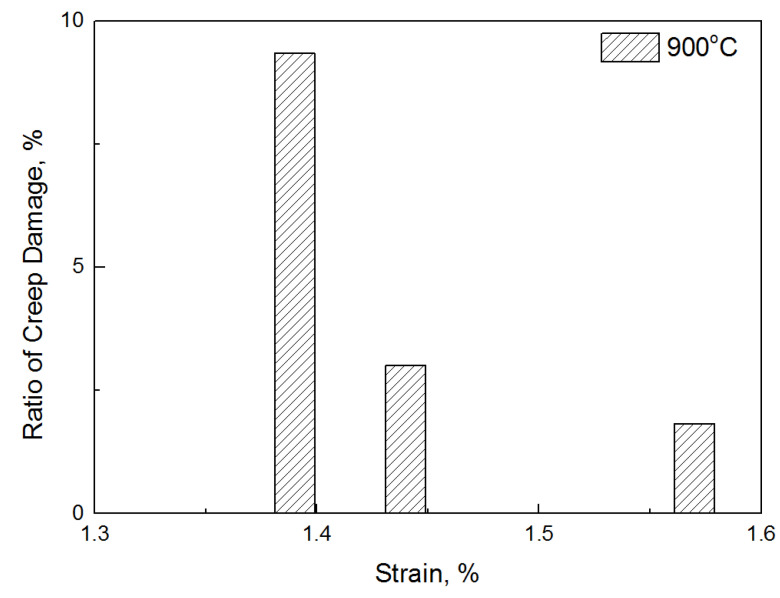
Ratio of damage caused by creep (ΣD_creep_) at 900°C.

In this study, an equation to prove that creep damage is caused by variable load was suggested, and the creep damage amount caused by low-cycle fatigue was derived quantitatively. Although Table 5 shows the amount of creep damage that occurred in only one cycle, the amount of creep damage that occurred throughout the low-cycle fatigue can also be derived by multiplying the fatigue life. Therefore, using the cumulative creep damage, the ratio of damage owing to creep in the fracture life of the total low-cycle fatigue is shown in Figure 10 and Figure 11. At 800 °C, the creep damage per cycle was the highest at 1.45%, which is the highest strain rate; however, the effect on the failure was approximately 1.8%. On the contrary, in the case of the lowest strain (1.05%), although the amount of creep damage that occurred per cycle was small, the percentage of damage caused by creep in the failure was approximately 9%. Similarly, at 900 °C, the highest strain (1.57%) had only a 1.8% effect on the failure; however, the lowest strain (1.39%) contributed to a failure of approximately 9.3%, which was eight times higher. In conclusion, a higher contribution to the fracture in the low strain was observed at both 800 °C and 900 °C, despite the low creep damage amount per cycle. This implies that the damage caused by creep occurring during fatigue is more affected by the number of repeated cycles than by the magnitude of the stress.

Considering that the amount of creep damage that occurred under variable load conditions performed in this study affected approximately 9% of the total life, the actual turbine operating environment may have a greater effect. Therefore, to predict the life of a turbine, it is necessary to consider the creep occurring under variable loads. In this study, only the amount of creep damage occurring under variable loads was derived. However, to predict the actual turbine life, it is necessary to consider variable load and steady-state creep damage. Therefore, if we rearrange Equation (2), which represents the cumulative damage law considering steady-state creep, we can obtain Equation (7) as follows:

(7) $$\sum D_{\mathrm{fatigue}}+\sum D_{\mathrm{creep}\left(\mathrm{variable}\;\mathrm{load}\right)}+\sum D_{\mathrm{creep}\left(\mathrm{steady}\;\mathrm{state}\right)}=1$$

Equation (7) can be arranged as Equation (8) by substituting Equation (6):

(8) $$D_{\mathrm{fatigue}}\cdot N_{f}+\left(\int_{k_{1}}^{\frac{P}{2}}\frac{1}{A{\left(f{\left(t\right)}_{\mathrm{ten}}\right)}^{n}}\mathrm{dt}+\int_{\frac{P}{2}}^{k_{2}}\frac{1}{A{\left(f{\left(t\right)}_{\mathrm{comp}}\right)}^{n}}\mathrm{dt}\right)\cdot N_{f}+\int_{0}^{t}\frac{1}{t_{r}}\mathrm{dt}=1$$

where t is the maintenance time of the steady state, and N_f_ is the fracture life of low-cycle fatigue. Equation (8) can be used to estimate the damage caused by pure fatigue; life can be predicted with respect to conditions, such as the number of startups and shutdowns, and the steady-state maintenance time. Life prediction and verification of low-cycle fatigue considering both variable loads and steady-state creep will be conducted in future research.

## 6. Conclusions

In this study, analysis was conducted on the amount of creep damage caused by variable loads. The method of deriving the amount of creep damage caused by variable loads was as follows:

1. By integrating the reciprocal of the creep rupture time with respect to time, the amount of creep damage occurring per unit time and the amount of creep damage occurring per cycle were derived;
2. The relationship between rupture time and stress was derived from the creep test result and expressed as a time integral expression for stress;
3. The stress change with time was derived from the fatigue test result, and the stress was expressed as a function of time;
4. The section where creep actually acted was classified according to the result of stress change over time in the fatigue test.

From the derived equation, the effect of creep occurring during the low-cycle fatigue test was analyzed. As a result of the analysis, as the temperature and strain increased, the amount of creep damage occurring during fatigue per fatigue cycle increased. However, based on the total failure, the creep damage increased as the strain decreased. Therefore, this indicates that the amount of creep damage that occurs under variable loads has a greater effect on the number of repetitions of fatigue than the magnitude of strain.

## Figures and Tables

**Table 1:** Chemical compositions of CMSX-4 (wt%).

Cr	Co	Mo	Re	W
6.5	9	0.6	3.0	6
**Al**	**Ti**	**Ta**	**Hf**	**Ni**
5.6	1.0	6.5	0.1	Rest

**Table 2:** Strain conditions during the low-cycle fatigue test.

	Strain		
800 °C	1.05%	1.31%	1.45%
900 °C	1.39%	1.44%	1.57%

**Table 3:** Value of parameters A and n.

	A (MPa^−n^·hour)	n
800 °C (R^2^ = 0.955)	1174	−0.074
900 °C (R^2^ = 0.973)	1200	−0.18

**Table 4:** Comparison of the coefficient of determination.

	Linear	2nd Poly	Exponential
800 °C	0.999513	0.99971	0.99977
900 °C	0.998953	0.99951	0.999593

**Table 5:** Creep damage per cycle at various temperature and strain values.

Temperature	Strain	D_creep_
800 °C	1.05%	1.885 × 10^−4^
	1.31%	1.893 × 10^−4^
	1.45%	1.896 × 10^−4^
900 °C	1.39%	2.616 × 10^−4^
	1.44%	2.711 × 10^−4^
	1.57%	2.853 × 10^−4^
